# *In silico* structure-based discovery of a SARS-CoV-2 main protease inhibitor

**DOI:** 10.7150/ijbs.59191

**Published:** 2021-04-10

**Authors:** Lei Wen, Kaiming Tang, Kenn Ka-Heng Chik, Chris Chun-Yiu Chan, Jessica Oi-Ling Tsang, Ronghui Liang, Jianli Cao, Yaoqiang Huang, Cuiting Luo, Jian-Piao Cai, Zi-Wei Ye, Feifei Yin, Hin Chu, Dong-Yan Jin, Kwok-Yung Yuen, Shuofeng Yuan, Jasper Fuk-Woo Chan

**Affiliations:** 1 State Key Laboratory of Emerging Infectious Diseases, Carol Yu Centre for Infection, Department of Microbiology, Li Ka Shing Faculty of Medicine, The University of Hong Kong, Pokfulam, Hong Kong Special Administrative Region, China.; 2 Key Laboratory of Tropical Translational Medicine of Ministry of Education, Hainan Medical University, Haikou, Hainan, China.; 3 Hainan-Medical University-The University of Hong Kong Joint Laboratory of Tropical Infectious Diseases, Hainan Medical University, Haikou, Hainan, and The University of Hong Kong, Pokfulam, Hong Kong Special Administrative Region, China.; 4 Department of Pathogen Biology, Hainan Medical University, Haikou, Hainan, China.; 5 School of Biomedical Sciences, Li Ka Shing Faculty of Medicine, The University of Hong Kong, Pokfulam, Hong Kong Special Administrative Region, China.; 6 Department of Clinical Microbiology and Infection Control, The University of Hong Kong-Shenzhen Hospital, Shenzhen, Guangdong Province, China.

## Abstract

The Coronavirus Disease 2019 (COVID-19) pandemic caused by the novel lineage B betacoroanvirus severe acute respiratory syndrome coronavirus 2 (SARS-CoV-2) has resulted in significant mortality, morbidity, and socioeconomic disruptions worldwide. Effective antivirals are urgently needed for COVID-19. The main protease (M^pro^) of SARS-CoV-2 is an attractive antiviral target because of its essential role in the cleavage of the viral polypeptide. In this study, we performed an *in silico* structure-based screening of a large chemical library to identify potential SARS-CoV-2 M^pro^ inhibitors. Among 8,820 compounds in the library, our screening identified trichostatin A, a histone deacetylase inhibitor and an antifungal compound, as an inhibitor of SARS-CoV-2 M^pro^ activity and replication. The half maximal effective concentration of trichostatin A against SARS-CoV-2 replication was 1.5 to 2.7µM, which was markedly below its 50% effective cytotoxic concentration (75.7µM) and peak serum concentration (132µM). Further drug compound optimization to develop more stable analogues with longer half-lives should be performed. This structure-based drug discovery platform should facilitate the identification of additional enzyme inhibitors of SARS-CoV-2.

## Introduction

Severe acute respiratory syndrome coronavirus 2 (SARS-CoV-2), the causative agent of the Coronavirus Disease 2019 (COVID-19) pandemic, is a novel lineage B betacoronavirus first discovered in Wuhan, China, in late 2019 1. SARS-CoV-2 is highly transmissible and rapidly disseminated worldwide to cause more than 102 million cases of COVID-19, including over 2.2 million deaths as of 2^nd^ February 2021 2-4. While the overall case-fatality rate of COVID-19 is about 2%, the infection is especially severe in the elderly and those with underlying diseases 4. In the past year, a number of potential antiviral treatments for COVID-19 have been evaluated in clinical trials. Examples include monotherapy and/or combinatorial regimen of remdesivir, interferon-β1b, lopinavir-ritonavir, and hydroxychloroquine 5-8. However, their effects on disease outcomes are restricted to selected groups of patients, and the interim results of the WHO Solidary Trial suggested that these treatments might have little or no effect on hospitalized COVID-19 patients in terms of the overall mortality, ventilation requirement, and duration of hospital stay 9. Therefore, discovery of additional effective antivirals for COVID-19 is urgently needed.

*De novo* development of new antiviral agents for emerging viral infections usually takes years and inevitably lags behind the rapid evolvement of the epidemics 10. To find immediately available treatment options for COVID-19, repurposing studies of existing drug compounds have been conducted 11. The major limitation of cell-based screening of antivirals is that it is highly laborious. An alternative strategy is to exploit *in silico* structure-based screening of chemical libraries which has the advantages of being fast and providing mechanistic insights related to the target viral protein structure 12.

Similar to other betacoronaviruses, including SARS-CoV and Middle East respiratory syndrome coronavirus (MERS-CoV), the genome of SARS-CoV-2 is arranged in the order of 5'-replicase [open reading frame (ORF) 1a/b]-structural proteins [Spike (S)-Envelope (E)-Membrane (M)-Nucleocapsid (N)]-3' 13, 14. The ORF1a/b encodes a number of viral enzymes with important roles in the viral replication cycle, including the main (M^pro^) or chymotrypsin-like cysteine (3CL^pro^) protease, papain-like cysteine protease (PL^pro^), RNA-dependent RNA polymerase (RdRp), and helicase, which are potentially druggable targets 10. The SARS-CoV-2 M^pro^ plays an important role in viral replication by processing polyproteins that are translated from viral RNA 13. The SARS-CoV-2 M^pro^ cleaves various non-structural proteins (nsp4 to nsp16), including the RdRp (nsp12) and helicase (nsp13). Because of its essential role in viral replication, the SARS-CoV-2 M^pro^ represents one of the most attractive antiviral drug targets 15, 16. A number of crystal structures of the SARS-CoV-2 M^pro^ with or without bound inhibitors have been recently reported 17-19. In this study, we established an *in silico* screening platform based on these crystal structures to identify potential SARS-CoV-2 M^pro^ inhibitors from a chemical library consisting >8,800 compounds.

## Materials and Methods

### Molecular docking

CovalentDock was used for covalent virtual screening of DrugBank compounds against SARS-CoV-2 M^pro^ 20, 21. Compounds with covalently bondable chemical groups (Michael acceptor and β-lactam family) were recognized with the scripts provided in the program package. The relevant parts of the ligand structure were altered, i.e., open-up of β-lactam ring or active C=C bond. Then a “dummy” atom was artificially attached to temporarily occupy the empty valence for covalent linkage with the receptor. The altered ligand structure was optimized with Amber GAFF forcefield during a short minimization 22. The crystal structure of M^pro^ (code: 6LU7) was retrieved from Protein Data Bank (PDB) 23. The charge/protonation state of protease protein was assigned with H++ server 24. Binding pockets on protein surface was defined according to the native ligand pose. The Sγ atom of the nucleophilic Cys145 in M^pro^ was assigned as the covalent linkage acceptor. Hbind was used to detect intermolecular hydrogen bonds and calculate SLIDE affinity score and direct hydrophobic contacts 25, 26. 3D intermolecular interaction plot was generated by Pymol.

### Main protease purification and enzymatic assay

Genes encoding the SARS-CoV-2 M^pro^ (residues 3264-3569) were cloned into the expression vector pETH. The recombinant proteins were expressed in *Escherichia coli* BL21(DE3) cells and purified using the Ni2+-loaded HiTrap Chelating System (GE Healthcare) according to the manufacturer's instructions. The purity of each protein was assessed by 12% sodium dodecyl sulfate-polyacrylamide gels (SDS-PAGE). The concentration of each protein was determined by using the Bicinchoninic Acid Protein Assay Kit (Sigma-Aldrich). The M^pro^ enzyme inhibition assay experiments were performed in triplicate in Greiner bio-one 96-well black microplates using the peptide substrate Dabcyl-KTSAVLQSGFRKM-E(Edans)-NH2 (GL Biochem) as previously described 27. The assay was performed in buffer composed of 20mM Tris base, 100mM NaCl, 1mM EDTA, 1mM DTT, pH 7.3, 50μM fluorescence substrate and 10μM M^pro^, with a final assay volume of 100μl. The compound was incubated with M^pro^ for 30min before addition of the substrate and fluorescence detection at emission 460nm and excitation 355nm. GC376 was included as a positive control inhibitor as previously reported 28.

### Virus strain and titration

A clinical isolate of SARS-CoV-2 HKU001a (GenBank accession number MT230904) obtained from a COVID-19 patient was used in this study 29. The virus was amplified by three additional passages in VeroE6 cells (American Type Culture Collection, ATCC) in DMEM medium supplemented with 1% fetal bovine serum (FBS, GibcoTM, Life Technologies Corporation, Massachusetts, USA) and 100units/ml penicillin plus 100μg/ml streptomycin to make working stocks of the virus (2×10^5^ 50% tissue culture infectious dose (TCID_50_)/ml) as previously described 29. For virus titration, aliquots of SARS-CoV-2 were applied on confluent VeroE6 cells in 96-well plates for TCID_50_ assay as previously described 30. Briefly, serial 10-fold dilutions of the virus were inoculated in a VeroE6 cell monolayer in quadruplicate and cultured in penicillin/streptomycin-supplemented DMEM and 1% FBS. The plates were observed for cytopathic effects for 4 days. Viral titer was calculated with the Reed and Münch endpoint method. One TCID_50_ was interpreted as the amount of virus that causes cytopathic effects in 50% of inoculated wells. All experiments with live SARS-CoV-2 was conducted in the Biosafety Level 3 facility of The University of Hong Kong 31-33.

### Cell lines and drug compounds

VeroE6 and Caco-2 cell lines were obtained from ATCC as we previously described 29, 34, 35. Trichostatin A was purchased from MedChemExpress (New Jersey, USA).

### Cytotoxicity assay

The 50% effective cytotoxic concentration (CC_50_) of trichostatin A in Caco-2 cells were determined by CellTiter-Glo® luminescent cell viability assay (Promega) as we previously described with slight modifications 11, 36-38. Briefly, Caco-2 cells (4×10^4^ cells/well) were incubated with different concentrations of trichostatin A for 48h, followed by the addition of 40µl/well of CellTiterGlo® substrate and detection of luminance after another 15 min. The CC_50_ was calculated using Sigma plot (SPSS) in an Excel add-in ED50V10.

### Viral load reduction assay

Viral load reduction assay was performed for the evaluation of antiviral potency 39. Briefly, SARS-CoV-2-infected (MOI = 0.01) Caco-2 cells were treated with different concentrations of drugs or dimethyl sulfoxide (DMSO) control. Then, cell culture supernatants were collected at 48hpi for viral RNA extraction and quantitative reverse transcription-polymerase chain reaction (qRT-PCR) as previously described with modifications 37, 40. The primers and probe sequences were against the RNA-dependent RNA polymerase/Helicase (RdRP/Hel) gene region of SARS-CoV-2: forward primer: 5'-CGCATACAGTCTTRCAGGCT-3'; reverse primer: 5'-GTGTGATGTTGAWATGACATGGTC-3'; specific probe: 5'-FAMTTAAGATGTGGTGCTTGCATACGTAGAC-IABkFQ-3'. The viral load reduction assay experiments were performed in triplicate and repeated twice for confirmation.

### Plaque reduction assay

Plaque reduction assay was performed to plot the half maximal effective concentration (EC_50_) as we previously described with slight modifications 41, 42. Briefly, VeroE6 cells were seeded at 4×10^5^ cells/well in 12-well tissue culture plates on the day before the assay was performed. After 24h of incubation, 50 plaque-forming units (PFU) of SARS-CoV-2 were added to the cell monolayer with or without the addition of drug compounds. The plates were further incubated for 1h at 37°C in 5% CO_2_ before removal of unbound viral particles by aspiration of the media and washing once with DMEM. Monolayers were then overlaid with media containing 1% low melting agarose (Cambrex Corporation, New Jersey, USA) in DMEM and appropriate concentrations of trichostatin A, inverted and incubated as above for another 72h. The wells were then fixed with 10% formaldehyde (BDH, Merck, Darmstadt, Germany) overnight. After removal of the agarose plugs, the monolayers were stained with 0.7% crystal violet (BDH, Merck) and the plaques counted. The percentage of plaque inhibition relative to the control (i.e. without the addition of compound) wells were determined for each drug compound concentration. EC_50_ was calculated using Sigma plot (SPSS) in an Excel add-in ED50V10. The plaque reduction assay experiments were performed in triplicate and repeated twice for confirmation.

### Immunofluorescence staining

Antigen expression in SARS-CoV-2-infected cells was detected with an in-house rabbit antiserum against SARS-CoV-2 nucleocapsid (N) protein as we previously described 43-45. Cell nuclei were labelled with the DAPI nucleic acid stain from Thermo Fisher Scientific (Waltham, MA, USA). The Alexa Fluor secondary antibody was obtained from Thermo Fisher Scientific. Mounting was performed with the Diamond Prolong Antifade mountant from Thermo Fisher Scientific. Imaging was taken and processed as we previously described 46.

### Time-of-drug-addition assay

Time-of-drug-addition assay was performed for trichostatin A as previously described with slight modifications 47. Briefly, VeroE6 cells were seeded in 24-well plates (2×10^5^ cells/well). The cells were inoculated with SARS-CoV-2 (MOI = 0.1) and then incubated for 1h for virus internalization. The viral inoculum was then removed and the cells were washed twice with PBS. At 0hpi (i.e. after PBS wash) and 3hpi, 10µM trichostatin A was added to the infected cells, followed by incubation at 37°C in 5% CO_2_ until 9hpi. For the “pre-incubation” time-point, 10µM trichostatin A was added to pre-treat cells at 2h before virus infection and then removed, followed by drug-free medium incubation with the cells until 9hpi. For the “co-infection” time-point, 10µM of trichostatin A was added together with the virus inoculation, followed by drug removal after 1h and incubation of the cells until 9hpi. At 9hpi, the cell culture supernatant of each time-point experiment was collected for viral load measurement using qRT-PCR. Dimethyl sulfoxide (0.5%) was included as a negative control for each group.

## Results

### Covalent virtual screening of SARS-CoV-2 M^pro^ inhibitors

**Figure 1:**
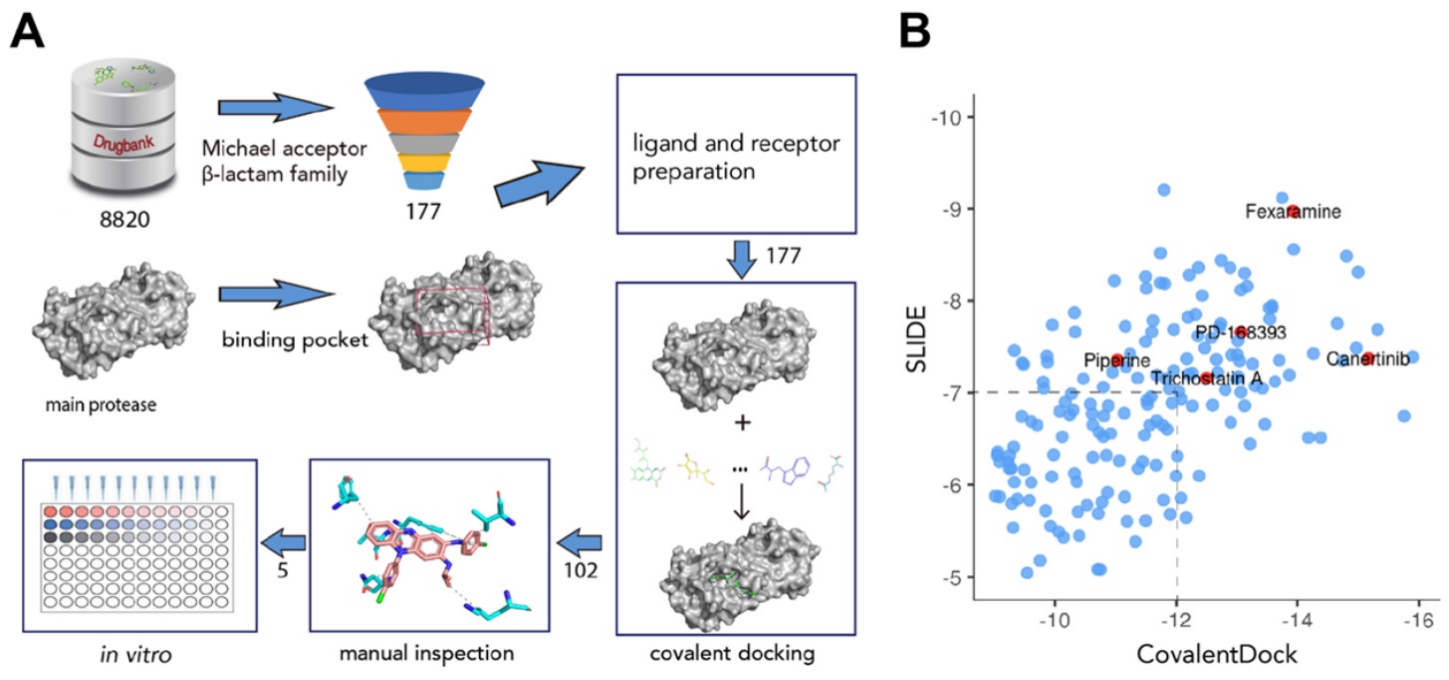
** Overview of the structure-based covalent docking virtual screening workflow.** (A) Small molecules in DrugBank database (8,820 compounds) were pre-filtered for covalent docking (Michael acceptor and β-lactam family). Potential binding pockets were predicted for SARS-CoV-2 M^pro^. Then each compound (177 candidates) was docked against binding pocket with CovalentDock. Binding poses (102 compounds) were manually inspected for downstream experimental validation based on certain criteria including relative binding affinity, ligand efficiency, hydrogen bond and hydrophobic contacts etc. (B) Scatter plot showing the distribution of CovalentDock and SLIDE scores of 177 docked poses. Five selected compounds were highlighted in red.

To screen for potential covalent inhibitors of SARS-CoV-2 M^pro^, DrugBank release version 5.1.6 which contains 8820 compounds with 3D structures available for docking was used for covalent virtual screening. Compounds with electrophilic chemical groups were first automatically recognized and prepared with CovalentDock (Figure 1). As a result, 177 compounds were selected for covalent docking screening. To eliminate pose scoring biases, SLIDE scoring function was also utilized for consensus scoring. A total of 75 drug compounds with CovalentDock score > -12 and SLIDE score > -7 were excluded, leaving 102 drug compounds for further analysis. After manual inspection and consideration of the hydrogen bond potential, shape complementarity of the binding pose, ligand efficiency, and hydrophobic contacts, 5 purchasable compounds, namely, canertinib, fexaramine, PD-168393, piperine, and trichostatin A, were selected as potential SARS-CoV-2 M^pro^ inhibitors for downstream experimental validation (Table S1).

### Trichostatin A inhibits SARS-CoV-2 M^pro^ activity *in vitro*

**Figure 2:**
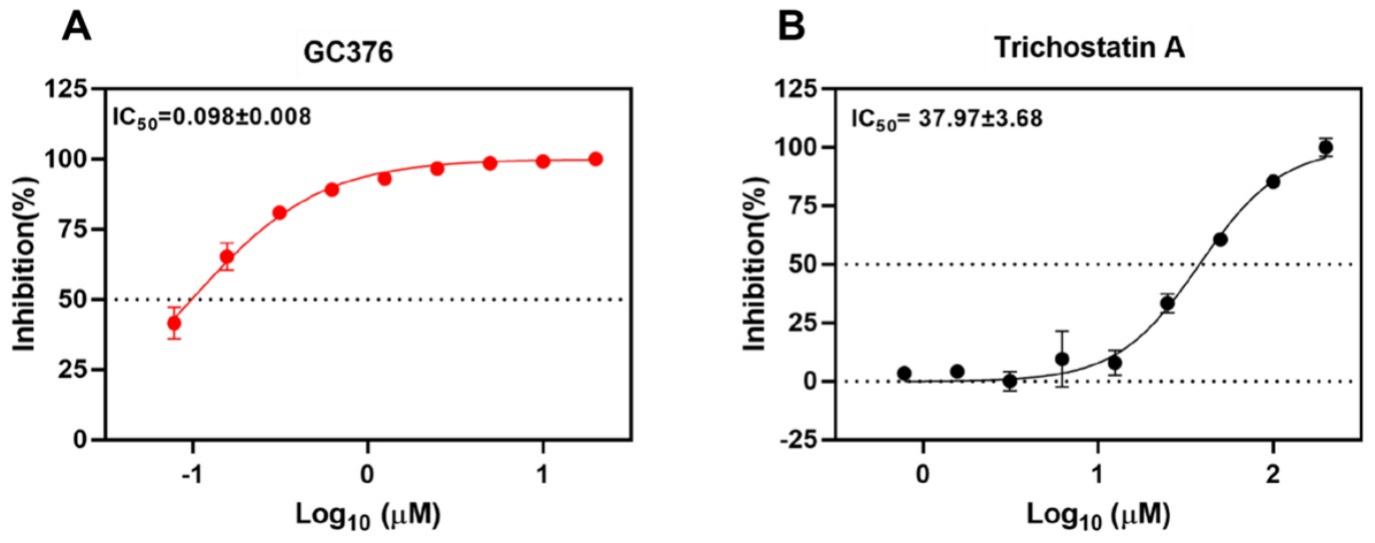
** Inhibition of the cleavage activity of SARS-CoV-2 M^pro^ by trichostatin A.** Titration of the protease activity of SARS-CoV-2 M^pro^ by (A) GC376 (positive control) and (B) trichostatin A at various concentrations as indicated by a fluorescence resonance energy transfer-based assay. The data were expressed as a percentage of the control reaction in the absence of inhibitors. Dose-response curves for half maximal inhibitory concentration (IC_50_) values were determined by nonlinear regression. The mean value of three replicates was shown and error bars indicated ±SD of n=3 independent replicates. All the experiments were repeated twice for confirmation.

To validate the SARS-CoV-2 M^pro^ inhibition of the selected drug compounds, we applied the EDANS-Dabcyl system for detection of M^pro^ cleavage activity and an anti-feline coronavirus drug compound GC376 with proven inhibitory activity against SARS-CoV-2 M^pro^ 28, 48. As expected, GC376 exhibited potent SARS-CoV-2 M^pro^ inhibition with an half maximal inhibitory concentration (IC_50_) of 0.098±0.008µM (Figure 2A). Among the 5 selected drug compounds, only trichostatin A showed reduction of M^pro^ activity in a dose-dependent manner (IC_50_ = 37.97±3.68µM) (Figure 2B). Therefore, we further evaluated the cytotoxicity and antiviral activity of trichostatin A with additional antiviral assays.

### Trichostatin A inhibits SARS-CoV-2 replication *in vitro*

**Figure 3:**
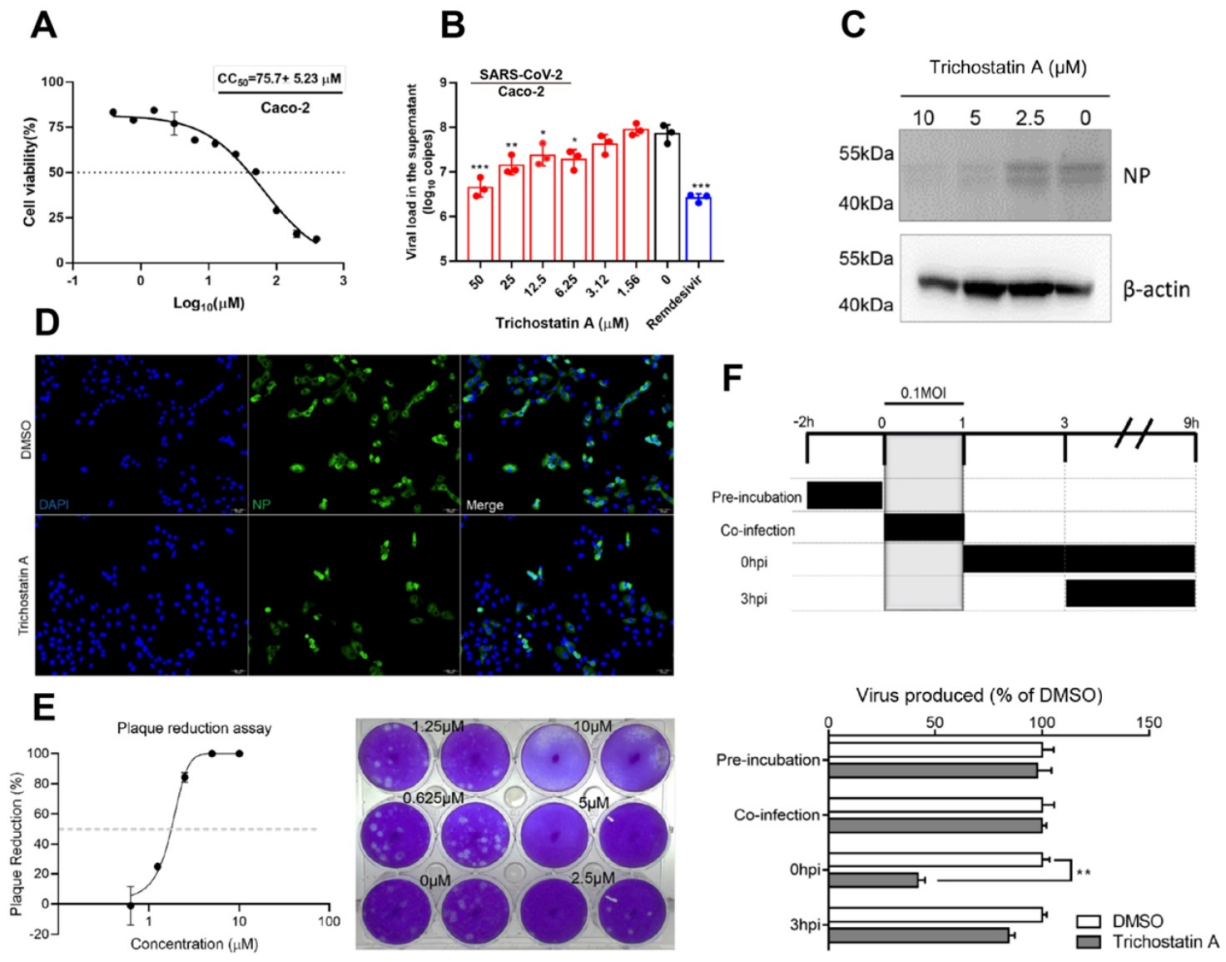
** Antiviral activity of trichostatin A against SARS-CoV-2.** (A) Cytotoxicity of trichostatin A in Caco-2 cells as determined by measuring the cellular ATP activity (CellTiter-Glo assay, 48h post drug treatment). (B) Multi-cycle virus growth assay in the presence or absence of trichostatin A. Caco-2 cells were infected with SARS-CoV-2 (MOI=0.01). Viral titers in cell culture supernatants were quantified by quantitative RT-PCR assay at 48hpi. Groups were analysed by One-way ANOVA when compared with the non-treated group (0µM). Remdesivir was included as a positive control. (C) Western blot showed reduced SARS-CoV-2 nucleocapsid protein production after trichostatin A treatment. Caco-2 cells with different treatments as indicated were infected with SARS-CoV-2 (MOI=0.1) and lysed at 24hpi. (D) Immunofluorescence staining of the SARS-CoV-2 nucleoprotein protein (green) and cell nucleus (blue). Fixation and staining were performed after trichostatin A (10µM) was used to treat SARS-CoV-2-infected (MOI=0.1) VeroE6 cells for 24h. (E) Plaque reduction assay showing the dose-dependent live SARS-CoV-2 reduction after trichostatin A treatment on VeroE6 cells. (F) Time-of-drug-addition assay. The upper panel depicts the scheme of experimental design; lower panel shows the viral titer collected in the cell culture supernatant and normalized by DMSO as a control. The experiments were performed in triplicate and replicated twice. The results are shown as mean± SD. * indicated P<0.05 and ** indicated P<0.01 (Student's t-test).

Trichostatin A is known as a histone deacetylase inhibitor and an antifungal antibiotic 49. The cytotoxicity and antiviral activity of trichostatin A were evaluated in Caco-2 cells as previously described 50. The CC_50_ of trichostatin A in Caco-2 cells was 75.7±5.2μM after 48h of incubation (Figure 3A). In the viral load reduction assay, trichostatin A (50μM) treatment reduced viral RNA load in Caco-2 cell culture supernatant by >1-log when compared with the DMSO control (EC_50_ = 2.7±0.8μM), resulting in a selectivity index (CC_50_/EC_50_) of 27.6. Similar inhibitory effect could be achieved in SARS-CoV-2-infected Caco-2 cells treated with 10µM of remdesivir (Figure 3B). Intracellularly, dose-dependent reduction of SARS-CoV-2 N protein production was detected in the cell lysate of trichostatin A-treated groups (Figure 3C). Moreover, immunofluorescence staining demonstrated marked suppression of SARS-CoV-2 N protein expression upon trichostatin A treatment (Figure 3D). To fully document the antiviral potency of trichostatin A, we further validated its anti-SARS-CoV-2 activity using plaque reduction assay. As shown in Figure 3D, 5μM and 10μM of trichostatin A completely inhibited plaque formation of SARS-CoV-2, resulting in an EC_50_ of 1.5±0.3μM (Figure 3E). Overall, we demonstrated that trichostatin A potently inhibited viral RNA load, antigen expression, and infectious particle formation of SARS-CoV-2 *in vitro* at non-cytotoxic concentrations.

### Trichostatin A interrupts the post-entry events of the SARS-CoV-2 replication cycle

To investigate the phase of the SARS-CoV-2 replication cycle interrupted by trichostatin A, we performed a time-of-drug-addition assay by exposing the virus-infected cells to the drug at different time-points during the viral replication cycle, followed by measurement of virus titers at 9 hours post-inoculation (hpi). VeroE6 cells were infected by 0.1 MOI of SARS-CoV-2 (Figure 3F). No significant inhibitory activity was observed when trichostatin A was added at the virus adsorption stage (0~1 hpi, termed “co-infection”). SARS-CoV-2 attachment was not affected when VeroE6 cells were pre-incubated with trichostatin A (-2 to 0hpi). Apparently, about 60% drop of progeny virions were detected when the drug was added after virus absorption (0 hpi), whereas the inhibitory effect became marginal when trichostatin A was added at 3 hpi. As progeny virus could be detected as early as 9 hpi, indicating completion of a single virus life cycle 51. Our time-of-drug-addition result suggested that trichostatin A interfered with the post-entry events of the SARS-CoV-2 replication cycle, which was compatible with the hypothesized role of trichostatin A as a SARS-CoV-2 M^pro^ inhibitor. The result also indicated that the proteolytic process executed by SARS-CoV-2 M^pro^ might occur with 3h after virus internalization.

### Potential binding mode of trichostatin A to the catalytic site of the SARS-CoV-2 M^pro^

**Figure 4:**
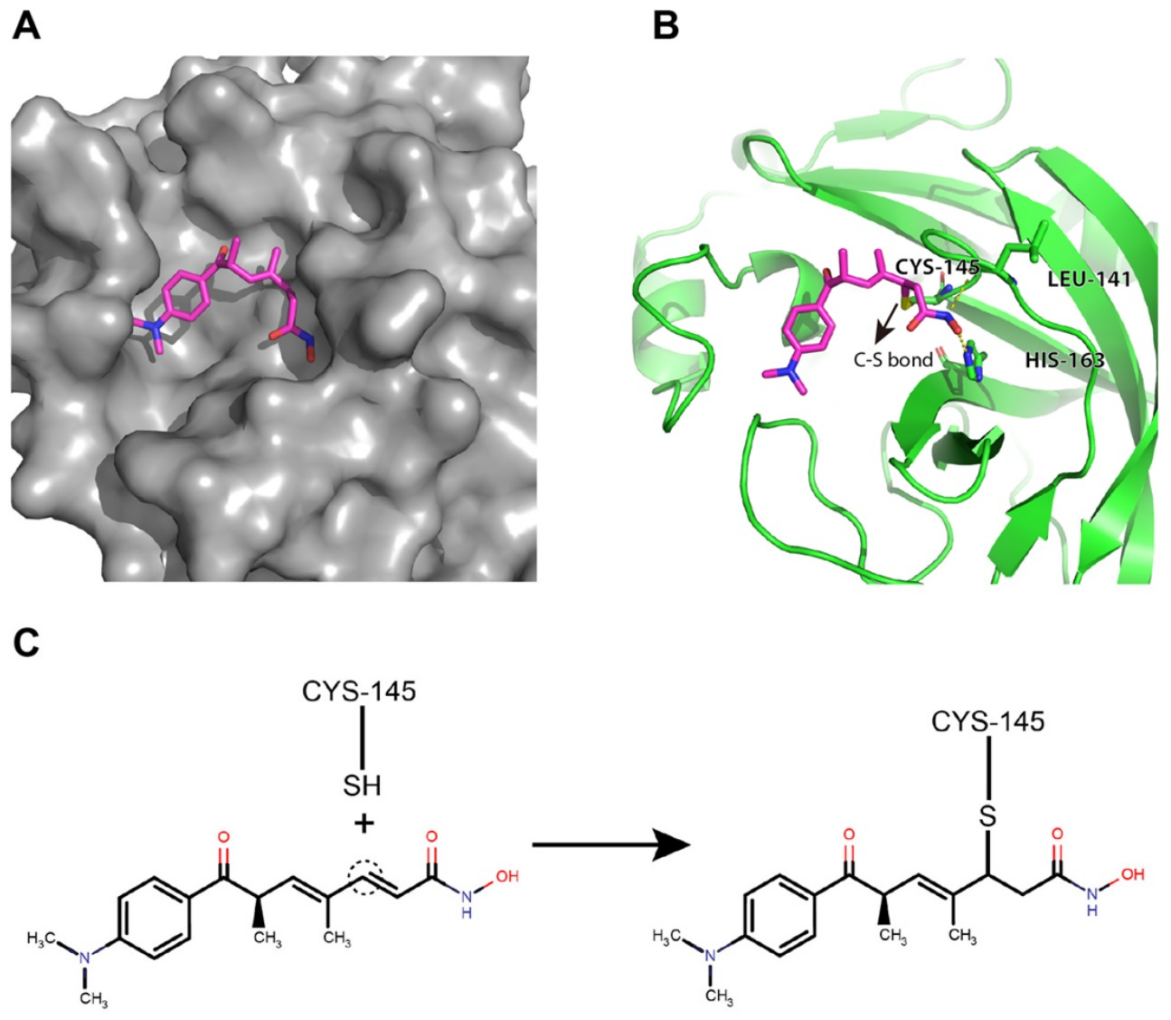
** Trichostatin A binds to the surface groove of SARS-CoV-2 M^pro^ with high stability.** (A) Trichostatin A is predicted to be embedded in the M^pro^ catalytic groove with good shape complementarity. (B) The cartoon and stick representation of binding mode of trichostatin A: the hydrogen bonds between trichostatin A and LEU-141 or HIS-163 were marked by yellow dashed lines. The C-S bond between trichostatin A and CYS-145 was also indicated (arrow). (C) Illustration of the covalent reaction between trichostatin A and CYS-145 thiol.

Trichostatin A was predicted to bind to the catalytic site of the SARS-CoV-2 M^pro^ with good shape complementarity (Figure 4A). When inspecting the hydrogen bonding potential, it was found that trichostatin A forms hydrogen bonds with LEU-141 backbone and HIS-163 sidechain to further stabilize the binding pose and improve the specificity (Figure 4B). Meanwhile, the ene-carbon of trichostatin A forms a covalent S-C linkage with the CYS-145 thiol (Figures 4B and 4C), which is a typical thiol Michael addition reaction (also known as thia-Michael addition). Thus, we reasoned that the complementary non-covalent interaction and the much stronger covalent linkage enabled trichostatin A to act as a potent SARS-CoV-2 M^pro^ inhibitor.

## Discussion

Protease inhibitors have been successfully used to treat viral infections clinically, including human immunodeficiency virus (HIV) and hepatitis C virus infections. We have also previously shown that novobiocin and bromocriptine might be repurposed as Zika virus NS2B-NS3 protease inhibitors 12, 51. For SARS, MERS, and COVID-19, we and others have demonstrated that the HIV protease inhibitor lopinavir was effective *in vitro* and/or *in vivo* 7, 10, 40, 52-55. In this study, we utilized *in silico* structure-based screening to identify potential SARS-CoV-2 M^pro^ inhibitors from a large chemical library consisting nearly 9,000 compounds. Among the primary hit compounds, we further validated trichostatin A's inhibitory effect of SARS-CoV-2 M^pro^ activity using an enzyme inhibition assay. The antiviral activity of trichostatin A against SARS-CoV-2 was evident in the drug's ability to significantly reduce the viral RNA load, viral antigen expression, and infectious virus particle formation. Corroborating with the expected role of M^pro^, our time-of-drug-addition assay showed that trichostatin A interrupted the post-entry events of the SARS-CoV-2 replication cycle. Molecular docking analysis predicted that trichostatin A was able to bind to the SARS-CoV-2 M^pro^ the catalytic site of with good shape complementarity.

Trichostatin A was originally reported as a fungistatic antibiotic obtained from a culture broth of *Streptomyces platensis* 56. Subsequently, its potent inhibitory effect on histone deacetylase (HDAC) activity was identified 56. Trichostatin A chelates zinc ions in the active site of HDAC which prevents histone unpacking and makes DNA less available for transcription. Trichostatin A selectively inhibits class I and II mammalian histone HDAC families of enzymes, but not class III HDACs 57. It is rapidly and extensively metabolized in mice following intraperitoneal administration, which is evidenced by its maximal serum concentration (C_max_) of 40 µg/ml (equivalent to 132µM) being achievable within 5 min of drug administration 58. Despite this rapid metabolism, the major metabolite of trichostatin A, N-Monomethyl trichostatin A amide, still exhibits HDAC inhibitory activity. The EC_50_ of trichostatin A against SARS-CoV-2 as determined by plaque reduction assay is around 1.5μM which is below the C_max_. Nevertheless, given the very short plasma half-life of trichostatin A (<10 minutes at 80mg/kg) 59, further drug compound optimization to develop more stable analogues with longer half-lives should be performed. Alternatively, synergistic effect between trichostatin A and other anti-SARS-CoV-2 drug compounds such as remdesivir, interferons, lopinavir, and ribavirin should be evaluated to identify potential combinatorial regimens in which trichostatin A may be used to enhance the effects of these clinically approved antivirals with longer half-lives.

Other drug compounds that have been reported to exhibit both inhibitory activity against the SARS-CoV-2 M^pro^ and reduced virus-induced cytopathic effects include the α-ketoamide boceprevir (antiviral EC_50_ = 1.31μM), the peptide-aldehyde calpain inhibitor II (antiviral EC_50_ = 2.07μM), and the sulfonate-featured peptide GC-376 (antiviral EC_50_ = 3.37μM) 60. Additionally, other drug compounds such as manidipine (anti-M^pro^ IC_50_ = 4.8μM), lercanidipine (anti-M^pro^ IC_50_ = 16.2μM), and bedaquiline (anti-M^pro^ IC_50_ = 18.7μM) have also demonstrated inhibitory activity against the SARS-CoV-2 M^pro^, but their effects against SARS-CoV-2 replication remain to be examined 61. Notably, boceprevir is an FDA-approved treatment for hepatitis C virus infection. Boceprevir (oral 800 mg three times daily) achieves a C_max_ of 1,723 ng/mL (equivalent to 3.3 µM), which is above its *in vitro* anti-SARS-CoV-2 IC_50_ (1.31μM) 62. GC-376 is an investigational drug with *in vivo* efficacy for treating cats with feline infectious peritonitis caused by feline coronavirus. GC-376 has a favourable C_max_ that is >100-fold of the *in vitro* EC_50_ against feline coronavirus and an elimination half-life (T_1/2_) of 3-5 hours 63. The *in vivo* efficacy of GC376 against mice infected with murine hepatitis virus or murine norovirus has also been reported 64.

The identification and validation of trichostatin A as a potent anti-SARS-CoV-2 drug compound has demonstrated the capability of our structure-based screening platform and *in vitro* enzyme inhibition and antiviral assays to discover SARS-CoV-2 M^pro^ inhibitors from a large chemical library. A similar approach could be adopted to screen additional libraries to find potential inhibitors of other key viral enzymes of SARS-CoV-2, including RdRp, helicase, and papain-like protease, to expand the treatment options for COVID-19.

## Supplementary Material

Supplementary table.

## References

[1] Zhou P, Yang XL, Wang XG, Hu B, Zhang L, Zhang W (2020). A pneumonia outbreak associated with a new coronavirus of probable bat origin. Nature.

[2] WHO (2021). Coronavirus disease (COVID-19): Weekly Epidemiological Update. [2 February 2021]. Available from: https://www.who.int/publications/m/item/weekly-epidemiological-update--2-february-2021.

[3] Chan JF, Yuan S, Kok KH, To KK, Chu H, Yang J (2020). A familial cluster of pneumonia associated with the 2019 novel coronavirus indicating person-to-person transmission: a study of a family cluster. Lancet.

[4] Li X, Sridhar S, Chan JF (2020). The Coronavirus Disease 2019 pandemic: how does it spread and how do we stop it?. Current opinion in HIV and AIDS.

[5] Beigel JH, Tomashek KM, Dodd LE, Mehta AK, Zingman BS, Kalil AC (2020). Remdesivir for the Treatment of Covid-19 - Final Report. The New England journal of medicine.

[6] Goldman JD, Lye DCB, Hui DS, Marks KM, Bruno R, Montejano R (2020). Remdesivir for 5 or 10 Days in Patients with Severe Covid-19. The New England journal of medicine.

[7] Hung IF, Lung KC, Tso EY, Liu R, Chung TW, Chu MY (2020). Triple combination of interferon beta-1b, lopinavir-ritonavir, and ribavirin in the treatment of patients admitted to hospital with COVID-19: an open-label, randomised, phase 2 trial. Lancet.

[8] Fiolet T, Guihur A, Rebeaud ME, Mulot M, Peiffer-Smadja N, Mahamat-Saleh Y (2021). Effect of hydroxychloroquine with or without azithromycin on the mortality of coronavirus disease 2019 (COVID-19) patients: a systematic review and meta-analysis. Clinical microbiology and infection.

[9] Pan H, Peto R, Henao-Restrepo AM, Preziosi MP, Sathiyamoorthy V, Abdool Karim Q (2020). Repurposed Antiviral Drugs for Covid-19 - Interim WHO Solidarity Trial Results. New England journal of medicine.

[10] Zumla A, Chan JF, Azhar EI, Hui DS, Yuen KY (2016). Coronaviruses - drug discovery and therapeutic options. Nature reviews Drug discovery.

[11] Riva L, Yuan S, Yin X, Martin-Sancho L, Matsunaga N, Pache L (2020). Discovery of SARS-CoV-2 antiviral drugs through large-scale compound repurposing. Nature.

[12] Yuan S, Chan JF, den-Haan H, Chik KK, Zhang AJ, Chan CC (2017). Structure-based discovery of clinically approved drugs as Zika virus NS2B-NS3 protease inhibitors that potently inhibit Zika virus infection in vitro and in vivo. Antiviral research.

[13] Chan JF, Kok KH, Zhu Z, Chu H, To KK, Yuan S (2020). Genomic characterization of the 2019 novel human-pathogenic coronavirus isolated from a patient with atypical pneumonia after visiting Wuhan. Emerging microbes & infections.

[14] Chan JF, Lau SK, To KK, Cheng VC, Woo PC, Yuen KY (2015). Middle East respiratory syndrome coronavirus: another zoonotic betacoronavirus causing SARS-like disease. Clinical microbiology reviews.

[15] Zhang LL, Lin DZ, Sun XYY, Curth U, Drosten C, Sauerhering L (2020). Crystal structure of SARS-CoV-2 main protease provides a basis for design of improved alpha-ketoamide inhibitors. Science.

[16] Ma CL, Sacco MD, Hurst B, Townsend JA, Hu YM, Szeto T (2020). Boceprevir, GC-376, and calpain inhibitors II, XII inhibit SARS-CoV-2 viral replication by targeting the viral main protease. Cell research.

[17] Zhang L, Lin D, Sun X, Curth U, Drosten C, Sauerhering L (2020). Crystal structure of SARS-CoV-2 main protease provides a basis for design of improved α-ketoamide inhibitors. Science.

[18] Lee J, Worrall LJ, Vuckovic M, Rosell FI, Gentile F, Ton AT (2020). Crystallographic structure of wild-type SARS-CoV-2 main protease acyl-enzyme intermediate with physiological C-terminal autoprocessing site. Nature communications.

[19] Jin Z, Du X, Xu Y, Deng Y, Liu M, Zhao Y (2020). Structure of M(pro) from SARS-CoV-2 and discovery of its inhibitors. Nature.

[20] Ouyang X, Zhou S, Su CTT, Ge Z, Li R, Kwoh CK (2013). CovalentDock: Automated covalent docking with parameterized covalent linkage energy estimation and molecular geometry constraints. Journal of Computational Chemistry.

[21] Wishart DS, Knox C, Guo AC, Cheng D, Shrivastava S, Tzur D (2008). DrugBank: a knowledgebase for drugs, drug actions and drug targets. Nucleic acids research.

[22] Case DA, Cheatham III TE, Darden T, Gohlke H, Luo R, Merz Jr (2005). KM, et al. The Amber biomolecular simulation programs. Journal of Computational Chemistry.

[23] Berman HM, Westbrook J, Feng Z, Gilliland G, Bhat TN, Weissig H (2000). The Protein Data Bank. Nucleic acids research.

[24] Gordon JC, Myers JB, Folta T, Shoja V, Heath LS, Onufriev A (2005). H++: a server for estimating pKas and adding missing hydrogens to macromolecules. Nucleic Acids Res.

[25] Zavodszky MI, Sanschagrin PC, Kuhn LA, Korde RS (2002). Distilling the essential features of a protein surface for improving protein-ligand docking, scoring, and virtual screening. Journal of computer-aided molecular design.

[26] Raschka S, Wolf AJ, Bemister-Buffington J, Kuhn LA (2018). Protein-ligand interfaces are polarized: discovery of a strong trend for intermolecular hydrogen bonds to favor donors on the protein side with implications for predicting and designing ligand complexes. Journal of computer-aided molecular design.

[27] Zhang L, Lin D, Sun X, Curth U, Drosten C, Sauerhering L (2020). Crystal structure of SARS-CoV-2 main protease provides a basis for design of improved alpha-ketoamide inhibitors. Science.

[28] Vuong W, Khan MB, Fischer C, Arutyunova E, Lamer T, Shields J (2020). Feline coronavirus drug inhibits the main protease of SARS-CoV-2 and blocks virus replication. Nature communications.

[29] Chu H, Chan JF, Yuen TT, Shuai H, Yuan S, Wang Y (2020). Comparative tropism, replication kinetics, and cell damage profiling of SARS-CoV-2 and SARS-CoV with implications for clinical manifestations, transmissibility, and laboratory studies of COVID-19: an observational study. Lancet Microbe.

[30] Chan JF, Yip CC, To KK, Tang TH, Wong SC, Leung KH (2020). Improved Molecular Diagnosis of COVID-19 by the Novel, Highly Sensitive and Specific COVID-19-RdRp/Hel Real-Time Reverse Transcription-PCR Assay Validated In Vitro and with Clinical Specimens. Journal of clinical microbiology.

[31] Chu H, Hu B, Huang X, Chai Y, Zhou D, Wang Y (2021). Host and viral determinants for efficient SARS-CoV-2 infection of the human lung. Nature communications.

[32] Yuan S, Chu H, Huang J, Zhao X, Ye ZW, Lai PM (2020). Viruses harness YxxØ motif to interact with host AP2M1 for replication: A vulnerable broad-spectrum antiviral target. Science advances.

[33] Yuan S, Chu H, Chan JF, Ye ZW, Wen L, Yan B (2019). SREBP-dependent lipidomic reprogramming as a broad-spectrum antiviral target. Nature communications.

[34] Chan JF, Yip CC, Tsang JO, Tee KM, Cai JP, Chik KK (2016). Differential cell line susceptibility to the emerging Zika virus: implications for disease pathogenesis, non-vector-borne human transmission and animal reservoirs. Emerging microbes & infections.

[35] Chan JF, Chan KH, Choi GK, To KK, Tse H, Cai JP (2013). Differential cell line susceptibility to the emerging novel human betacoronavirus 2c EMC/2012: implications for disease pathogenesis and clinical manifestation. Journal of infectious diseases.

[36] Tsang JO, Zhou J, Zhao X, Li C, Zou Z, Yin F (2021). Development of Three-Dimensional Human Intestinal Organoids as a Physiologically Relevant Model for Characterizing the Viral Replication Kinetics and Antiviral Susceptibility of Enteroviruses. Biomedicines.

[37] Yuan S, Chan JFW, Chik KKH, Chan CCY, Tsang JOL, Liang R (2020). Discovery of the FDA-approved drugs bexarotene, cetilistat, diiodohydroxyquinoline, and abiraterone as potential COVID-19 treatments with a robust two-tier screening system. Pharmacological research.

[38] Zhu Z, Chu H, Wen L, Yuan S, Chik KK, Yuen TT (2019). Targeting SUMO Modification of the Non-Structural Protein 5 of Zika Virus as a Host-Targeting Antiviral Strategy. International journal of molecular sciences.

[39] Yuan SF, Chan CCY, Chik KKH, Tsang JOL, Liang RH, Cao JL (2020). Broad-Spectrum Host-Based Antivirals Targeting the Interferon and Lipogenesis Pathways as Potential Treatment Options for the Pandemic Coronavirus Disease 2019 (COVID-19). Viruses.

[40] Yuan S, Chan CC, Chik KK, Tsang JO, Liang R, Cao J (2020). Broad-Spectrum Host-Based Antivirals Targeting the Interferon and Lipogenesis Pathways as Potential Treatment Options for the Pandemic Coronavirus Disease 2019 (COVID-19). Viruses.

[41] Chu H, Chan JF, Wang Y, Yuen TT, Chai Y, Shuai H (2020). SARS-CoV-2 Induces a More Robust Innate Immune Response and Replicates Less Efficiently Than SARS-CoV in the Human Intestines: An Ex Vivo Study With Implications on Pathogenesis of COVID-19. Cellular and molecular gastroenterology and hepatology.

[42] Chan JF, Zhang AJ, Yuan S, Poon VK, Chan CC, Lee AC (2020). Simulation of the Clinical and Pathological Manifestations of Coronavirus Disease 2019 (COVID-19) in a Golden Syrian Hamster Model: Implications for Disease Pathogenesis and Transmissibility. Clinical infectious diseases.

[43] Zhang AJ, Lee AC, Chu H, Chan JF, Fan Z, Li C (2020). SARS-CoV-2 infects and damages the mature and immature olfactory sensory neurons of hamsters. Clinical infectious diseases.

[44] Zhang AJ, Lee AC, Chan JF, Liu F, Li C, Chen Y (2020). Co-infection by severe acute respiratory syndrome coronavirus 2 and influenza A(H1N1)pdm09 virus enhances the severity of pneumonia in golden Syrian hamsters. Clinical infectious diseases.

[45] Yuan S, Wang R, Chan JF, Zhang AJ, Cheng T, Chik KK (2020). Metallodrug ranitidine bismuth citrate suppresses SARS-CoV-2 replication and relieves virus-associated pneumonia in Syrian hamsters. Nature microbiology.

[46] Yuan SF, Chu H, Huang JJ, Zhao XY, Ye ZW, Lai PM (2020). Viruses harness Yxxempty set motif to interact with host AP2M1 for replication: A vulnerable broad-spectrum antiviral target. Science advances.

[47] Yuan S, Chu H, Zhao H, Zhang K, Singh K, Chow BK (2016). Identification of a small-molecule inhibitor of influenza virus via disrupting the subunits interaction of the viral polymerase. Antiviral Res.

[48] Blanchard JE, Elowe NH, Huitema C, Fortin PD, Cechetto JD, Eltis LD (2004). High-throughput screening identifies inhibitors of the SARS coronavirus main proteinase. Chemistry & biology.

[49] You WJ, Steegborn C (2018). Structural Basis of Sirtuin 6 Inhibition by the Hydroxamate Trichostatin A: Implications for Protein Deacylase Drug Development. Journal of medicinal chemistry.

[50] Yuan SF, Chan JFW, Chik KKH, Chan CCY, Tsang JOL, Liang RH (2020). Discovery of the FDA-approved drugs bexarotene, cetilistat, diiodohydroxyquinoline, and abiraterone as potential COVID-19 treatments with a robust two-tier screening system. Pharmacol Res.

[51] Chan JF, Chik KK, Yuan S, Yip CC, Zhu Z, Tee KM (2017). Novel antiviral activity and mechanism of bromocriptine as a Zika virus NS2B-NS3 protease inhibitor. Antiviral Res.

[52] Chen F, Chan KH, Jiang Y, Kao RY, Lu HT, Fan KW (2004). In vitro susceptibility of 10 clinical isolates of SARS coronavirus to selected antiviral compounds. Journal of clinical virology.

[53] Chu CM, Cheng VC, Hung IF, Wong MM, Chan KH, Chan KS (2004). Role of lopinavir/ritonavir in the treatment of SARS: initial virological and clinical findings. Thorax.

[54] de Wilde AH, Jochmans D, Posthuma CC, Zevenhoven-Dobbe JC, van Nieuwkoop S, Bestebroer TM (2014). Screening of an FDA-approved compound library identifies four small-molecule inhibitors of Middle East respiratory syndrome coronavirus replication in cell culture. Antimicrobial agents and chemotherapy.

[55] Chan JF, Yao Y, Yeung ML, Deng W, Bao L, Jia L (2015). Treatment With Lopinavir/Ritonavir or Interferon-β1b Improves Outcome of MERS-CoV Infection in a Nonhuman Primate Model of Common Marmoset. The Journal of infectious diseases.

[56] Yoshida M, Matsuyama A, Komatsu Y, Nishino N (2003). From discovery to the coming generation of histone deacetylase inhibitors. Current medicinal chemistry.

[57] Vanhaecke T, Papeleu P, Elaut G, Rogiers V (2004). Trichostatin A - like hydroxamate histone deacetylase inhibitors as therapeutic agents: Toxicological point of view. Current medicinal chemistry.

[58] Sanderson L, Taylor GW, Aboagye EO, Alao JP, Latigo JR, Coombes RC (2004). Plasma pharmacokinetics and metabolism of the histone deacetylase inhibitor trichostatin A after intraperitoneal administration to mice. Drug metabolism and disposition.

[59] Salvador LA, Luesch H (2012). Discovery and mechanism of natural products as modulators of histone acetylation. Curr drug targets.

[60] Ma C, Sacco MD, Hurst B, Townsend JA, Hu Y, Szeto T (2020). Boceprevir, GC-376, and calpain inhibitors II, XII inhibit SARS-CoV-2 viral replication by targeting the viral main protease. Cell research.

[61] Ghahremanpour MM, Tirado-Rives J, Deshmukh M, Ippolito JA, Zhang CH, Cabeza de Vaca I (2020). Identification of 14 Known Drugs as Inhibitors of the Main Protease of SARS-CoV-2. ACS medicinal chemistry letters.

[62] Coilly A, Furlan V, Roche B, Barau C, Noël C, Bonhomme-Faivre L (2012). Practical management of boceprevir and immunosuppressive therapy in liver transplant recipients with hepatitis C virus recurrence. Antimicrobial agents and chemotherapy.

[63] Rathnayake AD, Zheng J, Kim Y, Perera KD, Mackin S, Meyerholz DK (2020). 3C-like protease inhibitors block coronavirus replication in vitro and improve survival in MERS-CoV-infected mice. Science Translational Medicine.

[64] Kim Y, Shivanna V, Narayanan S, Prior AM, Weerasekara S, Hua DH (2015). Broad-Spectrum Inhibitors against 3C-Like Proteases of Feline Coronaviruses and Feline Caliciviruses. Journal of virology.

