# Supplementary material for: In silico structure-based discovery of a SARS-CoV-2 main protease inhibitor
Source: Int J Biol Sci. 2021 Apr 10;17(6):1555–64. doi: 10.7150/ijbs.59191 (PMC8071767; doi:10.7150/ijbs.59191)
Supplement: Supplementary file 1 — Supplementary table. [file ijbsv17p1555s1.pdf]

ID	FORMULA	MOLECULAR WEIGHT	RBOND	NAME	GROUP	COVALENTDOCK	SLIDE	HBOND	CONTACTS
DB12259	C24H33N3O4	427.545	13	CG-200745	experimental	-16.02	-8.759	2	85
DB13164	C26H26N6O2S	486.59	7	Olmutinib	experimental	-15.9	-7.391	0	92
DB11907	C27H28F3N7O3	555.562	9	Rociletinib	experimental	-15.76	-6.75	0	66
DB15327	C26H26FN7O2	487.539	7	Abivertinib	experimental	-15.32	-7.692	1	64
DB05424	C24H25ClFN5O3	485.938	9	Canertinib	experimental	-15.17	-7.375	0	67
DB09330	C28H33N7O2	499.619	10	Osimertinib	approved	-15	-8.315	1	84
DB09053	C25H24N6O2	440.507	5	Ibrutinib	approved	-14.97	-7.487	1	66
DB15108	C31H33F3N2O5S	604.66	11	Tipranavir C-14	experimental	-14.81	-8.487	0	84
DB09211	C22H36O5	380.525	13	Limaprost	experimental	-14.75	-7.35	2	56
DB05616	C23H23N5O5	449.467	9	4'-Methylene-5,8,10-trideazaaminopterin	experimental	-14.66	-7.758	5	57
DB14768	C19H22ClN7O3	431.88	7	PF-06459988	experimental	-14.38	-6.512	1	48
DB08964	C23H38O5	394.552	13	Gemeprost	approved	-14.26	-7.427	1	71
DB07325	C17H19N5O4S	389.429	5	N-[(2-AMINO-6-METHYLPYRIMIDIN-4-YL)METHYL]-3-{[(E)-(2-OXODIHYDROFURAN-3(2H)-YLIDENE)METHYL]AMINO}BENZENESULFONAMIDE	experimental	-14.18	-6.512	3	33
DB12731	C24H29N3O2	391.515	8	Daporinad	experimental	-13.94	-8.561	1	73
DB02545	C32H36N2O3	496.6398	9	Fexaramine	experimental	-13.93	-8.975	3	76
DB06334	C22H19FN4O2	390.418	6	Tucidinostat	experimental	-13.87	-7.197	3	81
DB14850	C34H33BrN6O3	653.581	7	Deleobuvir	experimental	-13.75	-9.122	3	74
DB15170	C25H27N5O2	429.524	7	Evobrutinib	experimental	-13.7	-7.355	0	61
DB13101	C23H21N5O3S	447.51	5	4SC-202	experimental	-13.63	-7.124	1	77
DB00932	C31H33F3N2O5S	602.664	11	Tipranavir	approved	-13.59	-7.93	0	80
DB13019	C25H29FN4O4	468.529	5	Henatinib	experimental	-13.58	-7.947	3	60
DB01810	C23H24N2O5	408.4471	11	[1-(1-Methyl-4,5-Dioxo-Pent-2-Enylcarbamoyl)-2-Phenyl-Ethyl]-Carbamic Acid Benzyl Ester	experimental	-13.54	-7.805	1	78
DB08330	C19H18O3	294.3444	6	METHYL (2Z)-3-METHOXY-2-{2-[(E)-2-PHENYLVINYL]PHENYL}ACRYLATE	experimental	-13.53	-7.923	2	77
DB13342	C20H28N2O6	392.452	9	Cinepazet	experimental	-13.46	-6.663	1	60
DB06849	C22H24N2O2	348.4382	3	1-[1'-(3-phenylacryloyl)spiro[1-benzofuran-3,4'-piperidin]-5-yl]methanamine	experimental	-13.42	-7.05	3	46
DB13459	C17H25NO3	291.391	7	Pecilocin	experimental	-13.3	-7.076	1	65
DB15149	C22H22N6O3	418.457	6	Futibatinib	experimental	-13.26	-7.315	1	54
DB12428	C21H22F5N3O3S	491.48	8	PAC-14028	experimental	-13.22	-6.444	2	64
DB11741	C23H27FN4O2	410.493	6	Famitinib	experimental	-13.17	-8.164	4	57
DB06912	C15H20O4	264.3169	12	UNDECA-3,7-DIENE-1,3,7,11-TETRACARBALDEHYDE	experimental	-13.13	-6.861	1	57
DB11672	C21H20O6	368.3799	8	Curcumin	approved	-13.13	-8.304	2	82
DB05048	C28H38O6	470.606	11	Cannabinor	experimental	-13.07	-8.119	1	75
DB07662	C17H13BrN4O	369.215	4	PD-168393	experimental	-13.07	-7.663	0	58
DB07401	C22H17N3O5	403.3875	8	Azoxystrobin	experimental	-13.07	-7.007	1	49
DB08564	C20H20BrN5O	426.31	6	(2E)-N-{4-[(3-bromophenyl)amino]quinazolin-6-yl}-4-(dimethylamino)but-2-enamide	experimental	-13.05	-7.347	1	55
DB07559	C17H12Cl2N2O2	347.195	3	(2Z)-2-cyano-N-(2,2'-dichlorobiphenyl-4-yl)-3-hydroxybut-2-enamide	experimental	-13.03	-7.176	2	64
DB12123	C22H31N3O5	417.506	7	Cinepazide	experimental	-13.02	-7.566	1	58
DB08169	C19H16N2O2	304.3425	4	(2Z)-N-biphenyl-4-yl-2-cyano-3-cyclopropyl-3-hydroxyprop-2-enamide	experimental	-13.01	-7.416	1	66
DB07561	C19H18N2O3	322.3578	5	(2Z)-2-cyano-N-(3'-ethoxybiphenyl-4-yl)-3-hydroxybut-2-enamide	experimental	-13	-7.154	1	71
DB08172	C18H15ClN2O3	342.776	4	(2Z)-N-(3-chloro-2'-methoxybiphenyl-4-yl)-2-cyano-3-hydroxybut-2-enamide	experimental	-12.91	-7.412	1	55
DB06603	C21H23N3O2	349.434	7	Panobinostat	approved	-12.9	-6.681	0	60
DB05013	C25H34O6	430.541	4	Ingenol mebutate	approved	-12.89	-8.361	0	70
DB04376	C22H30O7	406.4694	3	13-Acetylphorbol	experimental	-12.81	-7.033	4	47
DB08435	C20H28O3	316.4345	11	(5E,14E)-11-oxoprosta-5,9,12,14-tetraen-1-oic acid	experimental	-12.74	-8.44	2	69
DB07500	C21H22O4	338.397	6	(2E)-1-[2-hydroxy-4-methoxy-5-(3-methylbut-2-en-1-yl)phenyl]-3-(4-hydroxyphenyl)prop-2-en-1-one	experimental	-12.73	-7.755	1	70
DB09236	C26H33NO6	455.551	11	Lacidipine	approved	-12.71	-7.714	1	72
DB12392	C16H19N3O4S	349.41	5	Resminostat	experimental	-12.68	-7.221	2	60
DB07350	C16H16N2O3	284.3098	5	(2E)-N-hydroxy-3-[1-methyl-4-(phenylacetyl)-1H-pyrrol-2-yl]prop-2-enamide	experimental	-12.66	-7.364	2	52
DB08125	C18H14N4O3S2	398.459	4	4-{[(2-Oxo-1,2-dihydro-3H-indol-3-ylidene)methyl]amino}-N-(1,3-thiazol-2-yl)benzenesulfonamide	experimental	-12.6	-6.868	0	57
DB06133	C23H24O6	396.439	9	Dimethylcurcumin	experimental	-12.58	-7.6	2	71
DB04297	C17H22N2O3	302.3682	6	Trichostatin A	experimental	-12.5	-7.162	2	51
DB12253	C26H20ClFN2O2	446.91	6	GDC-0810	experimental	-12.47	-7.487	1	69
DB12671	C29H41NO6	499.648	12	Beloranib	experimental	-12.43	-8.058	0	76
DB11942	C17H11F6N7O	443.313	7	Selinexor	approved	-12.38	-6.099	3	57
DB03104	C33H33N3O6	567.6316	10	2-[4-[(Z)-2-Acetamido-3-oxo-3-[[(3S)-2-oxo-1-[(4-phenylphenyl)methyl]azepan-3-yl]amino]prop-1-enyl]-2-formylphenyl]acetic acid	experimental	-12.37	-8.364	2	68
DB07775	C15H8Br2O6	444.028	1	3',5'-DIBROMO-2',4,4',6'-TETRAHYDROXY AURONE	experimental	-12.36	-7.619	5	47
DB12679	C17H14BrN3O	356.223	4	WP 1066	experimental	-12.32	-7.854	3	57
DB05524	C24H23ClFN5O2	467.93	8	Pelitinib	experimental	-12.27	-6.858	2	48
DB07443	C17H14N2O2	278.3053	3	(2Z)-N-biphenyl-4-yl-2-cyano-3-hydroxybut-2-enamide	experimental	-12.27	-7.214	1	64
DB05223	C20H30N4O2	358.486	10	Pracinostat	experimental	-12.22	-8.283	2	66
DB01268	C22H27FN4O2	398.4738	7	Sunitinib	approved	-12.21	-7.205	1	50
DB08009	C22H27ClN4O2	414.928	7	SU-11652	experimental	-12.2	-7.825	3	39
DB07078	C20H16N2O3	332.3526	3	(3Z)-6-(4-HYDROXY-3-METHOXYPHENYL)-3-(1H-PYRROL-2-YLMETHYLENE)-1,3-DIHYDRO-2H-INDOL-2-ONE	experimental	-12.18	-7.372	3	62
DB03445	C10H14N4O5S	302.307	8	(3S)-3-(dioxidosulfanyl)-N-[(1E)-3-oxoprop-1-en-1-yl]-4-(1H-1,2,3-triazol-1-yl)-D-valine	experimental	-12.17	-5.647	2	22
DB09496	C18H26O3	290.3972	10	Octinoxate	approved	-12.09	-6.932	0	72
DB03813	C14H25NO2S	271.419	11	2-Decenoyl N-Acetyl Cysteamine	experimental	-12.08	-5.858	0	65
DB06176	C24H36N4O6S2	540.69	2	Romidepsin	approved	-12.05	-7.012	1	46
DB02275	C13H15N3O3	261.2765	5	[2-Aminomethyl-5-Oxo-4-(4-Oxo-Cyclohexa-2,5-Dienylmethyl)-4,5-Dihydro-Imidazol-1-Yl] -Acetaldehyde	experimental	-12.02	-6.313	1	31
DB00903	C13H12Cl2O4	303.138	6	Etacrynic acid	approved	-12	-6.098	1	47
DB14122	C16H14O4	270.284	4	Dihydroxymethoxychalcone	experimental	-11.96	-7.242	2	54
DB08753	C21H21NO5	367.3951	9	4-{(1E)-3-OXO-3-[(2-PHENYLETHYL)AMINO]PROP-1-EN-1-YL}-1,2-PHENYLENE DIACETATE	experimental	-11.96	-7.692	2	46
DB08126	C16H15N5O3S	357.387	3	3-{[4-([amino(imino)methyl]aminosulfonyl)anilino]methylene}-2-oxo-2,3-dihydro-1H-indole	experimental	-11.93	-6.891	3	30
DB14924	C15H19N5O	285.351	3	PF-06651600	experimental	-11.9	-5.683	2	30
DB06741	C18H12Cl2N2O3	375.21	4	Gavestinel	experimental	-11.89	-7.043	3	55
DB12207	C18H12F6N6O	442.325	7	Verdinexor	experimental	-11.85	-6.603	3	66

DB15247	C23H26FN5O3	439.491	3	Vorolanib	experimental	-11.83	-7.156	2	63
DB02466	C24H26O3	362.4614	4	BMS-181156	experimental	-11.81	-8.19	2	58
DB05197	C27H30O6	450.5235	12	Sofalcone	experimental	-11.8	-9.211	4	89
DB05943	C15H17ClFNO4S	361.81	5	Resatorvid	experimental	-11.79	-5.836	0	48
DB06412	C21H32O3	332.484	0	Oxymetholone	approved	-11.78	-6.649	1	35
DB08407	C24H27NO7	441.4737	5	Platensimycin	experimental	-11.74	-8.199	5	75
DB07156	C21H20BrN3O2	426.306	4	(4Z)-6-bromo-4-({[4-(pyrrolidin-1-ylmethyl)phenyl]amino}methylidene)isoquinoline-1,3(2H,4H)-dione	experimental	-11.74	-8.518	1	63
DB07615	C18H17NO5	327.3313	6	Tranilast	experimental	-11.68	-7.194	2	49

DB11207	C15H20O3	248.322	7	Amiloxate	approved	-11.68	-6.7	1	56
DB14635	C21H20O9S	448.44	10	Curcumin sulfate	experimental	-11.67	-6.216	1	35
DB08754	C17H17NO4	299.3212	5	N-Caffeoyltyramine	experimental	-11.62	-7.789	3	52
DB01641	C17H19N5O4	357.3639	5	{(4Z)-2-[(1R,2R)-1-Amino-2-hydroxypropyl]-4-[(4-amino-1H-indol-3-yl)methylene]-5-oxo-4,5-dihydro-1H-imidazol-1-yl}acetic acid	experimental	-11.6	-7.665	4	50
DB06879	C21H22F2N6O	412.4358	2	5-(4'-AMINO-1'-ETHYL-5',8'-DIFLUORO-1'H-SPIRO[PIPERIDINE-4,2'-QUINAZOLINE]-1-YLCARBONYL)PICOLINONITRILE	experimental	-11.59	-6.959	2	46
DB08122	C16H15N3O3S	329.374	3	N-Methyl-4-{[(2-oxo-1,2-dihydro-3H-indol-3-ylidene)methyl]amino}benzenesulfonamide	experimental	-11.57	-6.653	2	32
DB03285	C15H12O4	256.2534	3	Isoliquiritigenin	experimental	-11.53	-6.876	2	44
DB07486	C21H18F6N2O2S	476.435	6	3-({4-[(1E)-3-morpholin-4-yl-3-oxoprop-1-en-1-yl]-2,3-bis(trifluoromethyl)phenyl}sulfanyl)aniline	experimental	-11.52	-6.919	4	40
DB00523	C20H28O2	300.4351	5	Alitretinoin	approved	-11.51	-8.138	3	68
DB12040	C12H19NO2	209.289	8	2-octyl cyanoacrylate	experimental	-11.5	-5.614	0	38
DB12326	C24H38O4	390.564	10	Antroquinonol	experimental	-11.5	-7.559	2	66
DB12029	C16H18O9	354.3087	5	Chlorogenic Acid	experimental	-11.5	-8.265	6	41
DB05015	C15H14N2O4S	318.35	4	Belinostat	approved	-11.48	-6.541	2	38
DB15086	C12H19NO2	209.2848	9	Ocrylate	experimental	-11.45	-6.253	1	35
DB15362	C25H17F3O4S	470.46	6	LSZ-102	experimental	-11.38	-6.18	2	27
DB11219	C18H22O	254.373	1	Enzacamene	approved	-11.37	-7.164	0	72
DB03834	C10H13N4O5S	301.3	8	(3S)-3-(dioxidosulfanyl)-N-[(1Z)-3-oxoprop-1-en-1-yl]-4-(1H-1,2,3-triazol-1-yl)-D-valine	experimental	-11.33	-5.383	3	32
DB05187	C22H24O4S	384.49	7	Elafibranor	experimental	-11.24	-7.425	1	53
DB06481	C15H11F3N2O2	308.26	5	Manitimus	experimental	-11.19	-5.606	0	57
DB11367	C16H19N3O5S	365.4	5	Cefroxadine	experimental	-11.17	-7.285	3	50
DB01720	C22H16BrNO4	438.271	6	(2Z)-2-(Benzoylamino)-3-[4-(2-bromophenoxy)phenyl]acrylic acid	experimental	-11.16	-7.726	1	49
DB06712	C19H19N3O6	385.3707	7	Nilvadipine	approved	-11.15	-5.873	1	48
DB15467	C14H18O4	250.294	8	Cinoxate	approved	-11.14	-6.781	2	43
DB06864	C11H12O6	240.2094	5	2-(3-CARBOXYPROPIONYL)-6-HYDROXY-CYCLOHEXA-2,4-DIENE CARBOXYLIC ACID	experimental	-11.06	-6.263	3	28
DB12582	C17H19NO3	285.3377	3	Piperine	experimental	-11.03	-7.358	0	58
DB11806	C24H32O2	352.518	4	VTP-194204	experimental	-10.98	-8.222	1	57
DB12072	C18H18N2O3	310.3471	4	Orantinib	experimental	-10.95	-7.227	1	44
DB08682	C16H18ClNO4S	355.836	5	1-METHYL ETHYL 1-CHLORO-5-[[(5,6DIHYDRO-2-METHYL-1,4-OXATHIIN-3-YL)CARBONYL]AMINO]BENZOATE	experimental	-10.92	-6.559	0	61
DB08110	C14H19NO4	265.305	4	(1R,4S,7AS)-1-(1-FORMYLPROP-1-EN-1-YL)-4-METHOXY-2,4,5,6,7,7A-HEXAHYDRO-1H-ISOINDOLE-3-CARBOXYLIC ACID	experimental	-10.88	-6.861	2	31
DB12223	C12H18O4	226.272	8	Squaric acid dibutyl ester	experimental	-10.87	-5.917	1	36
DB13297	C11H13NO2	191.23	4	Idrocilamide	experimental	-10.85	-6.321	2	39
DB07838	C17H12N2O4S2	372.418	4	(Z)-3-BENZYL-5-(2-HYDROXY-3-NITROBENZYLIDENE)-2-THIOXOTHIAZOLIDIN-4-ONE	experimental	-10.81	-6.752	0	60
DB11962	C18H11N3O2S	333.37	2	GSK-1059615	experimental	-10.8	-7.063	0	42
DB00265	C13H17NO	203.2802	3	Crotamiton	approved	-10.8	-6.523	0	39
DB12358	C8H11NO2	153.1784	5	Enbucrilate	experimental	-10.78	-5.814	1	30
DB12630	C9H13NO2	167.208	5	Isopentyl 2-cyanoacrylate	experimental	-10.75	-5.083	0	27
DB15359	C21H22O5	354.3964	6	Xanthohumol	experimental	-10.73	-6.572	2	33
DB07317	C11H11NO2	189.2105	2	(3E)-3-[(phenylamino)methylidene]dihydrofuran-2(3H)-one	experimental	-10.72	-5.691	0	31
DB06881	C6H11O6P	210.1217	5	(1Z)-2-HYDROXY-3-OXOHEX-1-EN-1-YL DIHYDROGEN PHOSPHATE	experimental	-10.71	-5.086	3	17
DB14783	C11H13NO6	255.226	7	Diroximel fumarate	approved	-10.63	-6.301	2	40
DB13063	C15H20O3	248.3175	0	Parthenolide	approved	-10.63	-6.655	0	41
DB07180	C17H16ClN3O2	329.781	2	5-[(Z)-(5-Chloro-2-oxo-1,2-dihydro-3H-indol-3-ylidene)methyl]-N,2,4-trimethyl-1H-pyrrole-3-carboxamide	experimental	-10.62	-6.774	2	31
DB01322	C14H16O3	232.275	3	Kava	approved	-10.59	-6.878	1	54
DB02892	C8H13NO4	187.1931	6	L-2-Amino-6-Methylene-Pimelic Acid	experimental	-10.53	-5.781	2	19
DB12150	C16H18O3	258.317	3	Pelubiprofen	experimental	-10.47	-7.156	1	52
DB08880	C12H9F3N2O2	270.2073	3	Teriflunomide	approved	-10.4	-5.447	1	41
DB04164	C13H17NO8	315.276	3	1,4-Deoxy-4-((5-Hydroxymethyl-2,3,4-Trihydroxycyclohex-5-Enyl)Amino)Fructose	experimental	-10.38	-5.708	3	31
DB07911	C12H9O4	217.1975	5	(3E)-2,6-DIOXO-6-PHENYLHEX-3-ENOATE	experimental	-10.36	-6.034	0	39
DB08039	C19H21N3O3S	371.453	2	(3Z)-N,N-DIMETHYL-2-OXO-3-(4,5,6,7-TETRAHYDRO-1H-INDOL-2-YLMETHYLIDENE)-2,3-DIHYDRO-1H-INDOLE-5-SULFONAMIDE	experimental	-10.33	-7.666	3	53
DB15590	C22H20O7	396.395	4	beta-Apopicropodophyllin	experimental	-10.32	-6.126	0	39
DB05171	C25H26FN3O2	419.5	5	E-2012	experimental	-10.32	-7.873	1	54
DB08396	C14H14N2O4	274.276	6	4-{[(Z)-(5-oxo-2-phenyl-1,3-oxazol-4(5H)-ylidene)methyl]amino}butanoic acid	experimental	-10.3	-6.813	4	31
DB12017	C13H12N2O2	228.251	4	Ozagrel	experimental	-10.27	-6.991	1	32
DB14175	C14H18O	202.2921	6	alpha-Amyl cinnamaldehyde	approved	-10.24	-6.764	1	38
DB05767	C20H30O5	350.455	3	Andrographolide	experimental	-10.17	-6.9	1	42
DB03211	C5H9O4P	164.0963	4	(3-Formyl-but-3-Enyl)-Phosphonic Acid	experimental	-10.14	-5.428	2	3
DB03626	C18H17NO4	311.3319	4	5-Methoxy-1,2-Dimethyl-3-(Phenoxymethyl)Indole-4,7-Dione	experimental	-10.12	-6.522	0	57
DB04227	C11H18N2O7	290.2698	5	9-Amino-2-deoxy-2,3-dehydro-n-acetyl-neuraminic acid	experimental	-10.08	-6.801	7	22
DB13331	C9H13NO2	167.208	2	Pyrithyldione	experimental	-10.06	-5.493	1	31
DB13214	C11H14N2O3S	254.3	2	Sulfadicramide	experimental	-9.98	-6.327	4	41
DB03903	C28H25N3O5	483.5152	5	Tmr	experimental	-9.96	-7.742	1	43
DB11285	C12H14O4	222.2372	5	Ethyl ferulate	approved	-9.95	-6.09	1	36
DB07819	C9H8ClNO2	197.618	2	(2E)-3-(4-CHLOROPHENYL)-N-HYDROXYACRYLAMIDE	experimental	-9.93	-5.553	0	26
DB00982	C20H28O2	300.4351	5	Isotretinoin	approved	-9.88	-7.334	0	55
DB06436	C15H14N2O	238.2845	1	Semaxanib	experimental	-9.87	-7.401	1	37
DB07818	C9H7Cl2NO2	232.063	2	(2E)-3-(2,4-DICHLOROPHENYL)-N-HYDROXYACRYLAMIDE	experimental	-9.85	-5.72	1	25
DB05974	C20H24O4	328.408	8	Transcrocetinate	experimental	-9.82	-7.049	1	40
DB15138	C25H25F3N2O2	442.482	5	AZD-9496	experimental	-9.81	-7.121	2	35
DB01793	C17H10Cl2N2O4	377.178	4	SB-409513	experimental	-9.76	-5.18	1	49
DB00755	C20H28O2	300.442	5	Tretinoin	approved	-9.71	-6.653	0	61
DB02400	C18H16N2O6	356.3294	5	ES-936	experimental	-9.61	-6.027	1	42
DB07978	C20H10F7NO4	461.2865	6	2-({[2,3,5,6-TETRAFLUORO-3'-(TRIFLUOROMETHOXY)BIPHENYL-4-YL]AMINO}CARBONYL)CYCLOPENTA-1,3-DIENE-1-CARBOXYLIC ACID	experimental	-9.61	-6.692	2	56
DB07529	C9H7N3OS	205.236	1	(5E)-2-Amino-5-(2-pyridinylmethylene)-1,3-thiazol-4(5H)-one	experimental	-9.58	-5.832	1	39
DB04085	C10H8N2O5	236.1809	4	Bis(N-maleimidomethyl)ether	experimental	-9.54	-5.045	0	24
DB07636	C14H17NO3S	279.35	6	5-Heptyl-6-hydroxy-1,3-benzothiazole-4,7-dione	experimental	-9.49	-6.166	0	64
DB02781	C16H15N2O4S	331.366	7	4-{(Z)-[2-[3-(Methylsulfanyl)Propanoyl]-5-Oxo-1-(2-Oxoethyl)-1,5-Dihydro-4h-Imidazol-4-Ylidene]Methyl}Benzenolate	experimental	-9.48	-7.31	1	43

DB08186	C13H13NO	199.2484	2	(3E)-4-(1-METHYL-1H-INDOL-3-YL)BUT-3-EN-2-ONE	experimental	-9.46	-7.328	3	41
DB04769	C12H7N3O2S	257.268	1	5-QUINOXALIN-6-YLMETHYLENE-THIAZOLIDINE-2,4-DIONE	experimental	-9.45	-6.747	1	36
DB13504	C14H15N5O5S2	397.42	5	Cefetamet	experimental	-9.38	-5.846	2	36
DB02816	C10H10N4O3S	266.276	3	7-(1-Methyl-1,2,3-Triazol-4-Yl)-6-Formyl-2,7-Dihydro-[1,4]Thiazepine-3-Carboxylic Acid, Brl42715, C6-(N1-Methyl-1,2,3-Triazolylmethylene)Penem	experimental	-9.33	-6.031	3	28
DB15201	C24H28O4	380.484	10	Norbixin	experimental	-9.33	-7.46	1	25
DB01332	C13H13N5O5S2	383.403	5	Ceftizoxime	approved	-9.32	-6.412	3	26
DB03428	C13H11N3O2	241.2453	2	SU9516	experimental	-9.31	-5.536	1	25
DB07516	C12H9ClO4	252.65	4	(2Z,4E)-3-chloro-2-hydroxy-6-oxo-6-phenylhexa-2,4-dienoic acid	experimental	-9.29	-5.798	1	46
DB04657	C12H13NO2S	235.302	2	Carboxin	approved	-9.28	-6.181	1	59
DB04347	C7H8O5	172.1354	1	3-Dehydroshikimate	experimental	-9.25	-6.179	4	24
DB03991	C11H17NO8	291.2546	5	2-deoxy-2,3-dehydro-N-acetylneuraminic acid	experimental	-9.25	-6.321	7	26
DB07960	C12H19NO6	273.2824	5	5-ACETAMIDO-5,6-DIHYDRO-4-HYDROXY-6-ISOBUTOXY-4H-PYRAN-2-CARBOXYLIC ACID	experimental	-9.23	-6.25	5	24
DB07503	C11H5F2NO4S	285.224	1	(5E)-5-[(2,2-DIFLUORO-1,3-BENZODIOXOL-5-YL)METHYLENE]-1,3-THIAZOLIDINE-2,4-DIONE	experimental	-9.19	-4.93	0	23
DB03321	C11H18N2O7	290.2698	5	Des(carbamimidoyl) zanamivir	experimental	-9.09	-6.255	6	18
DB08587	C11H12O5	224.21	4	Sinapic acid	experimental	-9.09	-5.871	1	21
DB03981	C6H8O9S	256.187	3	1,4-Dideoxy-5-Dehydro-O2-Sulfo-Glucuronic Acid	experimental	-9.06	-6.357	6	17
DB14184	C9H8O	132.1592	2	Cinnamaldehyde	approved	-9.05	-6.323	1	32
DB07788	C19H22O7	362.3738	1	(3R,5Z,8S,9S,11E)-8,9,16-TRIHYDROXY-14-METHOXY-3-METHYL-3,4,9,10-TETRAHYDRO-1H-2-BENZOXACYCLOTETRADECINE-1,7(8H)-DIONE	experimental	-9.02	-5.879	0	35
